# Preoperative proteinuria may be a risk factor for postoperative acute kidney injury：a meta-analysis

**DOI:** 10.1080/0886022X.2021.1940201

**Published:** 2021-06-21

**Authors:** Dan-Dan Huang, Yuan-Yuan Li, Zhe Fan, Yong-Gui Wu

**Affiliations:** Department of Nephropathy, The First Affiliated Hospital, Anhui Medical University, Hefei, Anhui, P. R. China

**Keywords:** Acute kidney injury, surgery, proteinuria, meta-analysis

## Abstract

**Objective:**

To investigate the relationship between preoperative proteinuria and postoperative acute kidney injury (AKI).

**Methods:**

We performed a search on databases included PubMed, Embase, the Cochrane Library, and Web of Science, from December 2009 to September 2020. Data extracted from eligible studies were synthesized to calculate the odds ratio (OR) and 95% confidence interval (CI). A fixed or random effects model was applied to calculate the pooled OR based on heterogeneity through the included studies.

**Results:**

This meta-analysis of 11 observational studies included 203,987 participants, of whom 21,621 patients suffered from postoperative AKI and 182,366 patients did not suffer from postoperative AKI. The combined results demonstrated that preoperative proteinuria is an independent risk factor for postoperative AKI (adjusted OR = 1.65, 95%CI:1.44–1.89, *p <* 0.001). Subgroup analysis showed that both preoperative mild proteinuria (adjusted OR = 1.30, 95%CI:1.24–1.36, *p* < 0.001) and preoperative heavy proteinuria (adjusted OR = 1.93, 95%CI:1.65–2.27, *p* < 0.001) were independent risk factors for postoperative AKI. The heterogeneity was combined because its values were lower. Further subgroup analysis found that preoperative proteinuria measured using dipstick was an independent risk factor for postoperative AKI (adjusted OR = 1.48, 95%CI:1.37–1.60, *p <* 0.001). Finally, preoperative proteinuria was an independent risk factor for postoperative AKI in the non-cardiac surgery group (adjusted OR = 2.06, 95%CI:1.31–3.24, *p* = 0.002) and cardiac surgery group (adjusted OR = 1.69, 95%CI:1.39–2.06, *p <* 0.001)

**Conclusion:**

Preoperative proteinuria is an independent risk factor for postoperative AKI and in instances when proteinuria is detected using dipsticks.

## 1. Introduction

Postoperative acute kidney injury (AKI) is a common complication that occurs after surgical procedures. The reported incidence of AKI after surgery ranges from 1 to 30% [1,2]. Postoperative AKI not only increases the cost and length of hospitalization, but also increases mortality [3–5]. However, effective treatment for this condition is yet to be established.

Postoperative AKI can be caused due to multiple reasons; hypovolemia, low systemic vascular resistance due to anesthesia or caval compression, and direct injury to the urinary system are all common events during surgery that can possibly harm the kidney. Factors that prolong the duration of renal ischemia and intraoperative hypotension during surgery are key determinants of postoperative AKI. In 2018, the perioperative quality initiative conducted a review of the literature from 1952 to 2017 and concluded that an intraoperative MAP below 60–70 mmHg was associated with an increased risk of AKI and mortality [6]. Therefore, despite the great advancements in surgical techniques, anesthetic methods, and critical care managements in the past decades, the incidence of postoperative AKI appears to have not changed significantly [7]. Recently, surgeons have begun to elucidate the relationship between preoperative risk factors and postoperative AKI.

Proteinuria has been reported to be associated with AKI in various clinical settings. Some studies have indicated that baseline proteinuria is an independent predictor for contrast-induced AKI [8,9]. Javier Neyra et al. evaluated 328 patients diagnosed with sepsis from January 2004 to June 2011, and found that proteinuria at admission was a significant predictor of AKI [OR = 2.3, 95% CI: 1.4–3.8, *p* < 0.001). In cardiac surgery, preoperative proteinuria has been shown to be associated with postoperative AKI regardless of the baseline renal function [10]. A meta-analysis demonstrates that there is a monotonous increase in adjusted hazard ratio of AKI associated with an increase in albuminuria across age, sex, and race [11]. However, the relationship between preoperative proteinuria and postoperative AKI in patients after surgery is currently unclear. Therefore, we have conducted a meta-analysis of studies to investigate the relationship between preoperative proteinuria and postoperative acute kidney injury.

## 2. Materials and methods

### 2.1. Search strategy of studies

Meta-analyses of study-level observational data were undertaken and reported according to the Preferred Reporting Items for Systematic Reviews and Meta-Analyses (PRISMA) guidelines [12]. Two independent reviewers (Yuanyuan Li and Zhe Fan) searched for observational research published from December 2009 to June 2020. They searched the electronic databases PubMed, Embase, the Cochrane Central Register, and Web of Science. The searching syntax included the following Medical Subject Heading (Mesh) and text words. The search strategy in Pubmed was as follows: (Preoperative Period [MeSH Terms]) OR (Preoperative Period [Title/Abstract]) OR (Period, Preoperative [Title/Abstract]) AND (Proteinuria [MeSH Terms]) OR (Proteinuria [Title/Abstract]) OR (Proteinurias [Title/Abstract]) AND (acute kidney injury [MeSH Terms]) OR (Acute Kidney Injuries [MeSH Terms]) OR (Kidney Injuries, Acute [MeSH Terms]) OR (Kidney Injury, Acute [MeSH Terms]) OR (Acute Renal Injury [MeSH Terms]) OR (Acute Renal Injuries [MeSH Terms]) OR (Renal Injuries, Acute [MeSH Terms]) OR (Renal Injury, Acute [MeSH Terms]) OR (Renal Insufficiency, Acute [MeSH Terms]) OR (Acute Renal Insufficiencies [MeSH Terms]) OR (Renal Insufficiencies, Acute[MeSH Terms]) OR (Acute Renal Insufficiency [MeSH Terms]) OR (Kidney Insufficiency, Acute [MeSH Terms]) OR (Acute Kidney Insufficiencies [MeSH Terms]) OR (Kidney Insufficiencies, Acute [MeSH Terms]) OR (Acute Kidney Insufficiency [MeSH Terms]) OR (Kidney Failure, Acute [MeSH Terms]) OR (Acute Kidney Failures [MeSH Terms]) OR (Kidney Failures, Acute [MeSH Terms]) OR (Acute Renal Failure [MeSH Terms]) OR (Acute Renal Failures [MeSH Terms]) OR (Renal Failures, Acute [MeSH Terms]) OR (Renal Failure, Acute [MeSH Terms]) OR (Acute Kidney Failure [MeSH Terms]) OR (acute kidney injury [Text Word]) OR (AKI [Title/Abstract] OR ARF[Title/Abstract]). In cases where important original data were missing, we contacted corresponding authors to obtain relevant data. We did not impute missing data. The complete search strategy is available in the Appendix.

### 2.2. Study eligibility criteria

The inclusion criterias are as follows: (a) proteinuria was tested using a dipstick or urine albumin to creatinine ratio (ACR). To classify the severity of proteinuria, we defined negative cases as “no proteinuria”, trace to 1 as “mild proteinuria”, and 2–4 as “heavy proteinuria”; (b）patients who met the AKIN OR 2012 KDIGO AKI diagnostic criteria and were postoperatively diagnosed with AKI; (c) original research article based on an epidemiologic study performed on humans; and (d) available information on the relationship between preoperative proteinuria and postoperative AKI.

Whereas the exclusion criterias are as follows: (a）no postoperative AKI; (b）no preoperative proteinuria; (c) no surgery; (d) case reports and reviews; (e) cross-sectional study; (f) note, letter, and Editorial; (g) conference abstract; (h) nephrectomy.

### 2.3. Data extraction and quality assessment

Two reviewers independently read the full text of the manuscripts and quality assessment, and data extraction are carried out independently, and disagreements are resolved through consensus. The trials in the meta-analyses included are defined by the 9-star Newcastle-Ottawa Scale [13] to evaluate the quality of the research, including selection, comparability, and exposure. Scores 1–3 were considered low quality, 4–7 were of moderate quality, and 8–11 were considered as high quality.

### 2.4. Statistical analysis

To investigate the relationship between preoperative proteinuria and postoperative AKI, the odds ratio (OR) of each study is calculated. The forest plot is used to describe the OR (95% CI). We assess heterogeneity among studies using the *I*^2^ statistics. When heterogeneity is lower (*I*^2^ < 50%), a Mantel–Haenszel fixed-effects model is applied for OR estimation. When heterogeneity is higher (*I*^2^>50%), a Mantel–Haenszel random-effects model is applied for OR estimation. Subgroup analysis is implemented based on the degree of preoperative proteinuria, different detection methods for preoperative proteinuria, and different types of surgery. Begger's funnel plot is carried out if the studies are more than ten. Egger's linear regression test is employed to check possible publication bias. The value of *p* is lower than 0.05 considered statistically significant. All statistical analyses are carried out using STATA 14 software (STATA Corp LP, College Station, Texas, USA).

## 3. Results

### 3.1. Study characteristics

**Figure 1.**
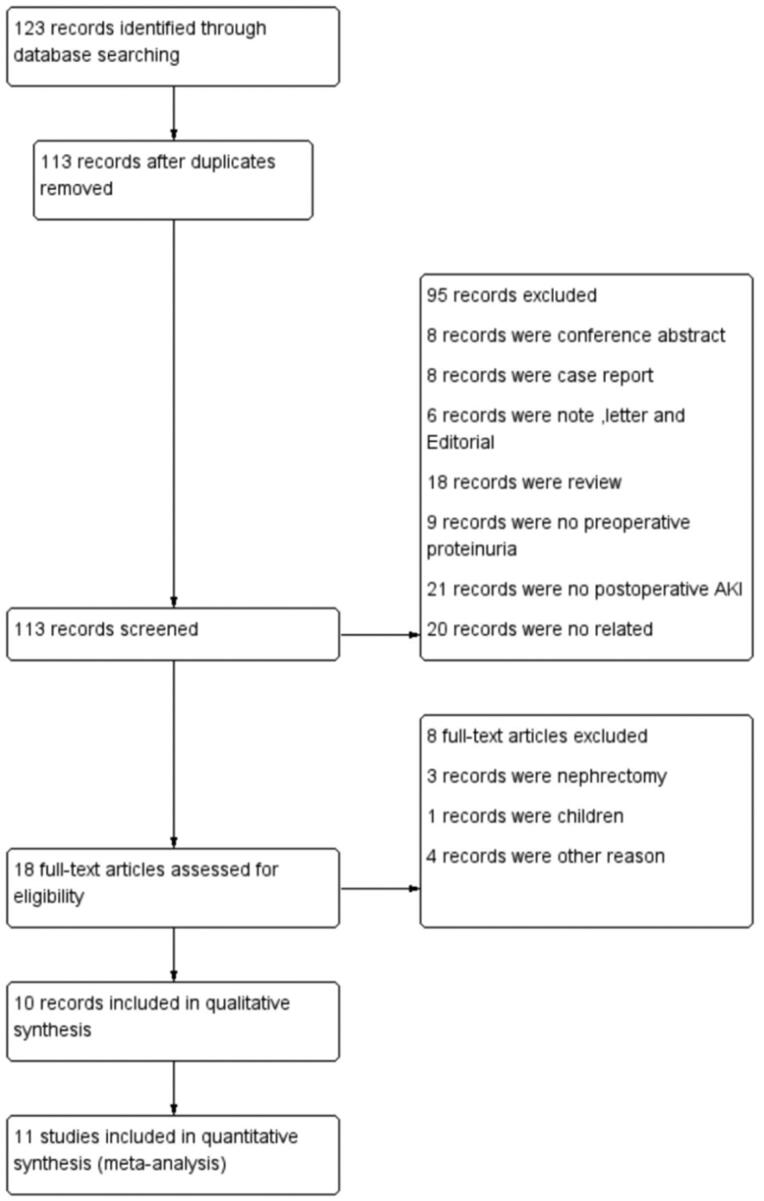
PRISMA flow diagram of the study.

The literature search is carried out on the PubMed, EMBASE, Cochrane Library, and Web of Science databases. We identify 123 records and use the information available in the title and abstract to filter the articles for the first time. The following are excluded: 10 duplicate records, 8 conference abstracts, 8 case reports, 6 notes, letters, or editorials, 18 reviews, 1 non-surgical record, 9 non-preoperative proteinuria records, 25 non-postoperative AKI records, 20 unrelated records, 1 record with children as participants, 3 nephrectomy records, and 4 records are excluded for other reasons. Therefore, we identify 10 records including 11 studies totally and report studies for our meta-analysis [14–24] (Figure 1).

The twelve studies include 203,987 participants, among whom 21,621 patients suffered from postoperative AKI and 182,366 patients do not. Among them, eleven studies evaluates the relationship between preoperative proteinuria and postoperative AKI, six studies investigates the relationship between the degree of preoperative proteinuria and postoperative AKI, eight studies uses the dipstick method to detect proteinuria, two studies uses the urine ACR to detect proteinuria, six studies investigates the relationship between preoperative proteinuria and postoperative AKI in cardiac surgery, four studies evaluates the relationship between preoperative proteinuria and postoperative AKI in non-cardiac surgery (Table 1).

**Table 1.** The characteristics of included studies in meta-analysis.

Author	Year	Design	Number of subjects	Definition Of AKI	Detection of proteinuria	HBP	DM	Mild proteinura	Heavy proteinura	AKI	Protein AKI	No AKI	Protein No AKI	Type of surgery	NOS score
N. Safai	2008	Prospectively study	398	A rise of more than 50% above baseline in serum creatinine on the postoperative day 3 or 5	Dipstick					44	4	354	41	Cardiac surgery	3-2-3
Tao-Min Huang,	2011	Prospectively study	1052	AKIN	Dipstick	66.5%	41.2%	315	138	183	123	868	330	CABG	3-2-3
Rezan Aksoy	2019	Retrospective study	634	KDIGO	Dipstick	54.73%	100%			230	35	404	38	CABG	3-2-3
Steven G. Coca (a),	2012	Prospectively study	1123	AKIN	Dipstick	78.86%	41.33%	316	87	450	110	673	81	Cardiac surgery	3-2-3
Steven G. Coca (b),	2012	Prospectively study	1159	AKIN	ACR	78.86%	41.33%	684	70	467	206	692	193	Cardiac surgery	3-2-3
Sehoon Park	2017	Retrospective study	40090	the international AKI guidelines.	Dipstick	47.56%	9.11%	1808	1226	2582		37508		All surgery	4-2-3
Tyler S. Wahl,	2018	Retrospective study	153767	KDIGO	Dipstick	70.42%	27.12%	56279	10917	17209	10428	136558	56768	noncardiac surgery	4-2-3
Masatoshi Nishimoto	2019	Retrospective cohort study.	5168	KDIGO	Dipstick	35.37%	15.65%	287	195	309		4859		noncardiac surgery	3-2-3
Jin-Tae Kwon	2018	Retrospective study	210	KDIGO	Dipstick	60%	52.86%			85	19	125	12	CABG	3-2-3
Fei He	2012	Retrospective case control	325	AKIN	Not mentioned	77.54%	32.31%			51	31	274	36	intracoronary stent implantation	2-2-3
Diamantina Marouli	2018	Retrospective study	61	AKIN	ACR	60.66%	31.14%			11	6	50	9	Major Abdominal Surgery	3-2-3

AKIN：acute kidney injury network; KDIGO: kidney disease improving global outcome; ACR: albumin to creatinine ratio; HBP: high blood pressure; DM: diabetes mellitus; AKI: acute kidney injury; CABG: coronary artery bypass grafting; NOS: Newcastle-Ottawa Scale.

### 3.2. Article quality

Of the ten articles, nine records are cohort studies and one record is a case-control study. One article score 7 points, seven articles scores 8 points and two articles scores 9 points in the literature quality evaluation (Table 1).

### 3.3. Meta-analysis of result measurement

#### 3.3.1. The relationship between preoperative proteinuria and postoperative AKI

**Figure 2.**
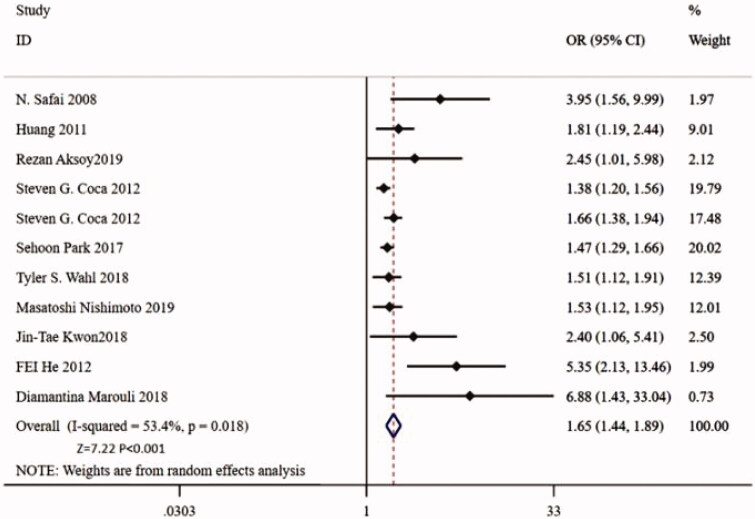
Forest plot for the relationship between preoperative proteinuria and postoperative acute kidney injury.

Eleven studies reports the relationship between preoperative proteinuria and postoperative AKI. The combined results demonstrate that preoperative proteinuria is an independent risk factor for postoperative AKI (adjusted OR = 1.65, 95% CI: 1.44–1.89, *p <* 0.001, *p* for heterogeneity = 0.018, and *I*^2^ =53.4) (Figure 2).

### 3.4. Subgroup analyses

#### 3.4.1. Degree of proteinuria

**Figure 3.**
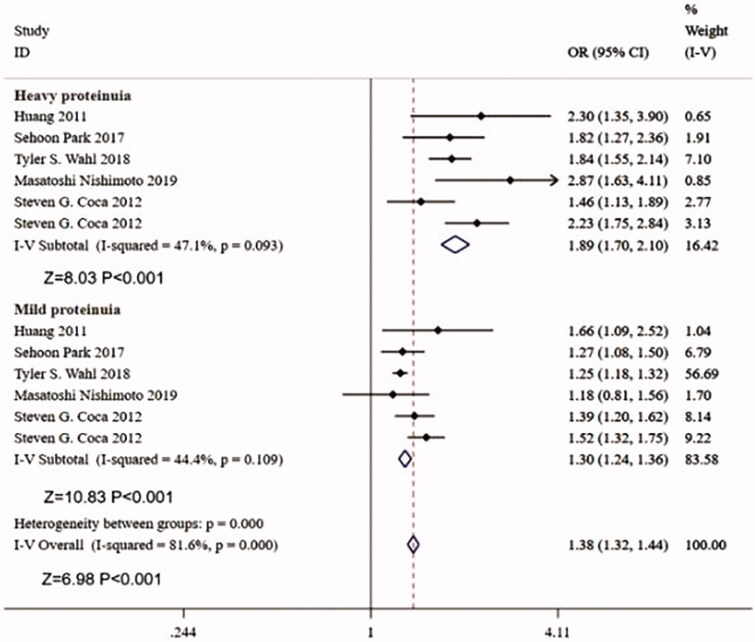
Forest plot for the subgroup analysis between preoperative proteinuria and postoperative acute kidney injury on the degree of proteinuria.

Seven studies investigates the relationship between the degree of preoperative proteinuria and postoperative AKI. Subgroup analysis shows that both heavy proteinuria (adjusted OR = 1.89, 95%CI:1.70–2.14, *p* < 0.001, *p* for heterogeneity = 0.093, and *I*^2^=47.1) and mild proteinuria (adjusted OR = 1.30, 95%CI:1.24–1.36, *p* < 0.001, *p* for heterogeneity = 0.109, and *I*^2^=44.4) are independent risk factors for postoperative AKI (Figure 3).

#### 3.4.2. Detection methods for proteinuria

**Figure 4.**
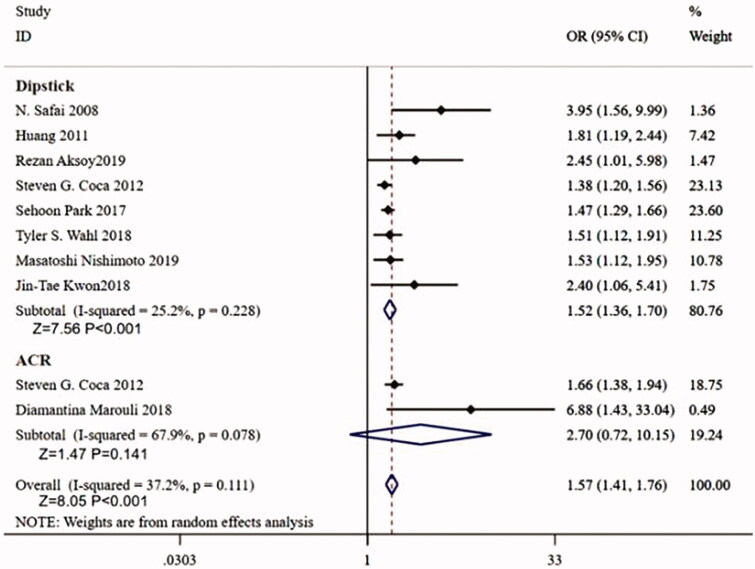
Forest plot for the subgroup analysis between preoperative proteinuria and postoperative acute kidney injury on the detection methods for proteinuria.

In the dipstick detection group, proteinuria is an independent risk factor for postoperative AKI (adjusted OR = 1.52, 95%CI:1.36–1.70, *p <* 0.001, *p* for heterogeneity = 0.228, and *I*^2^=25.2), while in the ACR detection group, proteinuria is not an independent risk factor for postoperative AKI (adjusted OR = 2.70, 95%CI 0.72–10.15, *p =* 0.141, *p* for heterogeneity = 0.078, and *I*^2^=67.9) (Figure 4).

#### 3.4.3. Types of surgery

**Figure 5.**
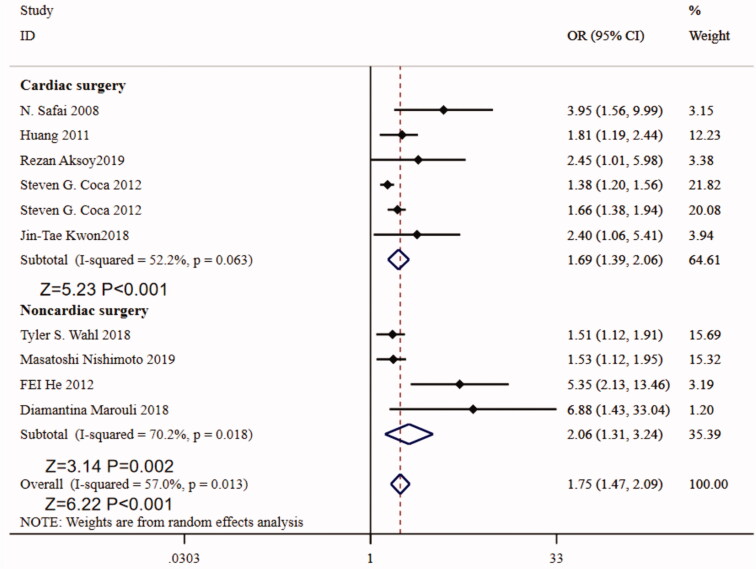
Forest plot for the subgroup analysis between preoperative proteinuria and postoperative acute kidney injury on the types of surgery.

In the non-cardiac surgery group, preoperative proteinuria is an independent risk factor for postoperative AKI (adjusted OR = 2.06, 95%CI:1.31–3.24, *p =* 0.002, *p* for heterogeneity = 0.018, and *I*^2^=70.2), in the cardiac surgery group, proteinuria is also an independent risk factor for postoperative AKI (adjusted OR = 1.69, 95%CI 1.39–2.06, *p <* 0.001, *p* for heterogeneity = 0.063, and *I*^2^=52.2) (Figure 5).

### 3.5. Funnel plot

**Figure 6.**
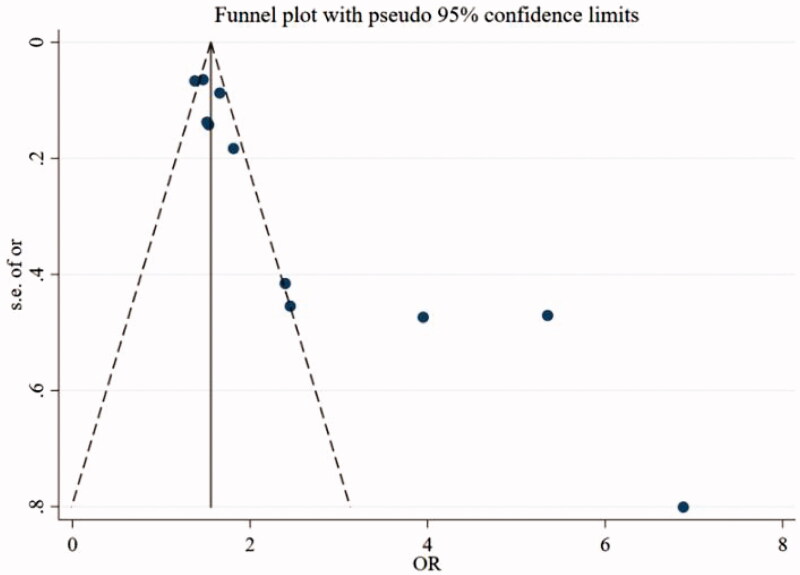
Funnel plot for the meta-analysis of the relationship between preoperative proteinuria and postoperative acute kidney injury.

The funnel plot is suggested by the Egger’s linear regression test. There are some publication bias observed on the relationship between preoperative proteinuria and postoperative AKI (*p* = 0.001 < 0.05) (Figure 6).

## 4. Discussion

Postoperative AKI is a common complication of surgical operation, which occurred due to a variety of factors. It includes the surgery timing (e.g., emergency surgery), proper intraoperative hemodynamic monitoring and management, and early postoperative intervention [25]. Chronic vascular disease, chronic kidney disease, hypertension, cardiac failure, and diabetes, aging 56 years or older, smoking history, which are the recently reported risk factors related to postoperative AKI [26].

Proteinuria is a common clinical manifestation of chronic kidney disease, as well as its relationship with AKI has also been confirmed by more and more studies. The pathophysiological explanation of the association between proteinuria independent of baseline glomerular filtration rate (GFR) and risk factors of primary diseases, especially the existence of AKI by hypertension or diabetes mellitus (DM). The first possibility is that proteinuria is thought to be caused by acute kidney infection or immune inflammation. Secondly, the possibility is that proteinuria can lead to hypoalbuminemia, and then the decrease of inflation pressure caused by hypoalbuminemia lead to the contraction of intravascular volume and susceptibility to AKI. In our meta-analysis, the comprehensive results demonstrate that preoperative proteinuria is an independent risk factor for postoperative AKI.

As we all know, different degrees of proteinuria mean that patients are in different pathological states. Those who with severe proteinuria are more likely to have DM, hyperuricemia, higher creatinine level and lower estimated-GFR level, which indicates that the kidney is more vulnerable to damage. However, recent studies have found that neutrophil gelatinase-associated lipocalin (NGAL) and kidney injury molecule-1are up-regulated in damaged renal tubules, which have been confirmed to predict AKI and unrelated to the degree of proteinuria. In our meta-analysis, no matter the degree is mild or heavy, patients with preoperative proteinuria have a higher risk of postoperative AKI. The incidence of AKI in preoperative mild proteinuria group and preoperative heavy proteinuria group is low heterogeneous, so we combined them.

Dipstick proteinuria and urine ACR are two methods to detect proteinuria. In subgroup analysis, dipstick proteinuria is an independent risk of postoperative AKI, while urine ACR is not. The detection limit of dipstick protein is 150 mg/L, and the clinical ACR ≥ 30 mg/g is the diagnostic standard of early diabetic nephropathy. The detection of dipstick protein is easy to detect, and it is not affected by urine pH and urine specific gravity [27]. Recent studies showed that urine ACR was related to vascular endothelial damage [28]. Endothelial dysfunction can lead to a decrease in microvascular blood flow and an increase in blood viscosity, leading to cellular ischemia and hypoxia, and increase inflammation [29]. It is an important indicator of renal damage. However, the denominator of urine ACR is urine creatinine, and the detection method is affected by the daily total creatinine production. When acute kidney injury occurs, the daily blood creatinine and urine creatinine change greatly. Moreover, in patients with larger muscle mass, the creatinine excretion may be much higher than 1000 mg/d, and the urine ACR will underestimate the proteinuria level; while in patients with consumptive constitution or with lower muscle mass, the creatinine excretion may be much lower than 1000 mg/d, and the urine ACR level will overestimate the proteinuria level [30]. The detection of urine ACR is also a kind of source of heterogeneity. So the urine protein detection with dipstick is more accurate.

There are many risk factors that can lead to acute kidney injury after operation. The incidence of AKI after cardiac surgery is as high as 30% [31]. Subgroup analysis showed that preoperative proteinuria is an independent risk factor for AKI in both cardiac surgery and non-cardiac surgery. The previous findings suggested that preoperative proteinuria within two days before surgery was highly predictive of postoperative AKI, irrespective of acute or chronic insults, which was confirmed by Wang [32]. However, different types of surgery have different design schemes, different affecting factors, different surgical procedures and different outcomes, which can increase heterogeneity, especially in non-cardiac surgery.

The advantages of our meta-analysis include comprehensive search, large sample size, and inclusion of the latest research with high quality of methodology. The result in this research is a suggestion both in scientific viewpoint and in clinical practice. Our meta-analysis have some limitations. First of all, the number of included researches was not abundant and there was publication bias. Secondly, ten studies were cohort studies and one was case-control study. Methodological differences can increase heterogeneity. Thirdly, the method of meta-analysis is collect the values of OR or RR from the logistic regression model by the adjustment of other risk factors for each study, and then use them to analyze. However, different studies are adjusted for different risk factors. Most of the studies are adjusted for at least age, sex, race, diabetes, heart failure and drugs. Besides, for instance, Coca’s study also adjusted for the type of operation and Nishimoto1’s study not only adjusted for the type of operation, but also adjusted for the minimum systolic blood pressure (SBP) during operation, SBP triangle during operation. These will also increase heterogeneity.

## 5. Conclusion

Proteinuria is closely associated with the progression of renal function. Recent studies have found that preoperative proteinuria is associated with postoperative renal function progression, postoperative cardiovascular complications, and the need for renal replacement therapy [33]. In this meta-analysis of observational study on patients undergoing surgery, preoperative proteinuria may be an independent risk factor for postoperative AKI and in instances when the detection of proteinuria is dipstick. Due to the serious heterogeneity, we need more randomized controlled trials to confirm it.
